# Estimation of salivary cortisol among subjects undergoing dental extraction

**DOI:** 10.4317/jced.54369

**Published:** 2018-02-01

**Authors:** Srikanth Gadicherla, Revathi-Panduranga Shenoy, Bhavik Patel, Meenakshi Ray, Brijesh Naik, Kalyana-Chakravarthy Pentapati

**Affiliations:** 1 Department of Oral and Maxillofacial Surgery, Manipal College of Dental Sciences, Manipal Academy of Higher Education, Manipal; 2 Department of Biochemistry, Kasturba Medical College, Manipal Academy of Higher Education, Manipal; 3 Deparment of Public Health Dentistry, Manipal College of Dental Sciences, Manipal Academy of Higher Education, Manipal

## Abstract

**Background:**

Dental procedures can be stressful and studies have shown that salivary cortisol is elevated during such procedures. Our study aimed to evaluate saliva cortisol levels among the subjects who underwent dental extractions and to compare it with that of the controls. The secondary objective of the study was to evaluate any correlation between salivary cortisol and hemodynamic parameters.

**Material and Methods:**

We conducted this clinical study among subjects, who were indicated for dental extraction. Saliva samples from the subjects in the study group were collected before and after (10 mins) the dental extraction. Hemodynamic parameters like heart rate, systolic (SBP) and diastolic blood pressure (DBP) and oxygen saturation (Sp O2) were measured 10 minutes prior to the dental extraction and after completion of the extraction by a single trained examiner. Salivary cortisol was estimated by solid phase enzyme-linked immunosorbent assay (ELISA).

**Results:**

A total of 31 subjects in the study group and 24 subjects in control group have participated in this study. The mean salivary cortisol concentration was significantly higher after extraction (27.94±7.94) than before extraction (24.67±8.31) in the study group (*P*<0.001). No significant correlations were seen between salivary cortisol concentration and hemodynamic parameters except for diastolic blood pressure after extraction.

**Conclusions:**

Dental extractions and local anaesthetic procedures can induce stress in subjects. Dental care providers should try to minimise the subject’s anxiety and stress to the maximum extent.

** Key words:**Cortisol, dental, extraction, saliva, stress.

## Introduction

Stress is a biological phenomenon which defies definition as it is a highly subjective experience. When the human body is challenged, stress leads to the activation of the autonomic nervous system and the hypothalamic-pituitary-adrenal (HPA) axis (1). Autonomic nervous system activates the “fight or flight” response through sympathetic nervous system while the parasympathetic nervous system returns the body to homeostasis (1). On the other hand, HPA axis alters the release of cortisol from the adrenal cortex (2). Cortisol is known to regulate many bodily functions which include psychological, metabolic and immunological. When the body is stressed, reactive cortisol levels begin to rise in body fluids (2). Salivary cortisol levels are known to highly correlate with serum cortisol levels and have many advantages such as non-invasive sample collection and a high acceptability among the patients (3). Studies conducted previously, have depicted elevated salivary cortisol levels as a measure of stress during various dental procedures like extractions (4,5) and administration of local anesthesia (6) and in subjects who suffer from dental pain (7). However, minor oral surgical procedures like dental extractions, which are common procedures carried out by dental care providers, have not been studied extensively (4). The stress, incurred by the individual during a routine dental extraction is substantial and two fold, due to the administration of local anaesthesia as well as the removal of the tooth. Although some procedures performed in the dental office are capable of unnerving the patient, distress related to injection of an anaesthetic is one of the most common psychological responses. The stress before the injection of anaesthetic produces more fear than the actual injection. Previous studies have shown, the increase in systolic and diastolic blood pressure among individuals who underwent extraction regardless of the anaesthetic used, with or without vasoconstrictor, while no significant difference was seen with respect to pulse rate (8).

Therefore, a study evaluating the level of stress during dental extraction would be of great value. In addition, the hemodynamic changes that occur due to a surge in stress, are also an important measure. Heart rate, blood pressure and oxygen saturation are important measures to check for the body’s response to stress, including chances of hypoxia. Hence, we aimed to evaluate saliva cortisol levels among the subjects who underwent dental extractions and to compare it with that of the control subjects. The secondary objective of the study was to evaluate any correlation between salivary cortisol with hemodynamic parameters.

## Material and Methods

We conducted this clinical study among subjects, who were indicated for dental extraction in the out-patient department of Oral and Maxillofacial Surgery. Participants who were 18 years or above undergoing dental extractions, under local anaesthesia were included in the study group. Subjects with syndromes, medical co-morbidities or comprehension problems, patients with altered saliva secretion due to any medications, local infection or swelling or pus discharge were excluded. In the control group, subjects who were 18 years or above, not undergoing any minor oral surgical procedure were included. The study protocol was approved by Institutional Ethics Committee of Manipal University, Manipal. The protocol was registered with clinical trial registry of India (REF/2017/05/014297). Prior informed consent was sought from all the subjects.

Prior to the collection of saliva, subjects were asked to rinse their mouth with plain water to clear away any debris or food. After five minutes, they were asked to spit saliva (approximately 1-2ml) in a sterile plastic container. Saliva samples from the subjects in the study group were collected before and after (10 mins) the dental extraction. Care was taken to ensure non-contamination of saliva with blood. All the samples were collected from 9 to 11 AM to avoid any variations due to circadian rhythms. Collection of saliva (MR) during the dental extractions (GS) were done by single trained calibrated experimenters. Each sample of saliva was labelled and stored at -20˚C until further biochemical analysis for cortisol. Hemodynamic parameters like heart rate, systolic (SBP) and diastolic blood pressure (DBP) and oxygen saturation (Sp O2) were measured 10 minutes prior to the dental extraction and after completion of the extraction by a single trained examiner (BP). For subjects in the control group, saliva collection and hemodynamic parameters were recorded only once.  

-Estimation of salivary cortisol 

Salivary cortisol was estimated by solid phase enzyme-linked immunosorbent assay (ELISA) (DRG Internationals, USA) in ng/ml. The procedure was done as per the manufacturer guidelines on thawed saliva samples and optical density was read at 450nm.

-Statistical analysis

All the analysis was done using SPSS version 18. A *P*-value of <0.05 was considered statistically significant. Comparison of mean salivary cortisol before and after dental extraction in the study group was done using the paired t-test. Comparison of mean salivary cortisol, before and after dental extraction of the study group with the control group was done using student’s t-test. Pearson’s correlation coefficient was done to evaluate the correlation between cortisol and hemodynamic parameters before and after the dental extraction.  

## Results

A total of 31 subjects in the study group and 24 subjects in control group have participated in this study. All the subjects in the study group underwent single simple extractions without any complications. The mean age in study and control group were 46.26±18.86 and 40.54±15.38 years respectively (*P*=0.233). A total of 16 and 11 subjects were females in study and control groups respectively (*P*=0.671). The mean salivary cortisol concentration was significantly higher after extraction (27.94±7.94) than before extraction (24.67±8.31) in the study group (*P*<0.001) ( Table 1). The salivary cortisol in the study group before (24.67±8.31) and after (27.94±7.94) dental extraction was significantly higher than the control group (14.42±2.49) (*P*<0.001 and <0.001) respectively ( Table 2). No significant correlations were seen between salivary cortisol concentration and hemodynamic parameters except for diastolic blood pressure after extraction ( Table 3).

**Table 1:**
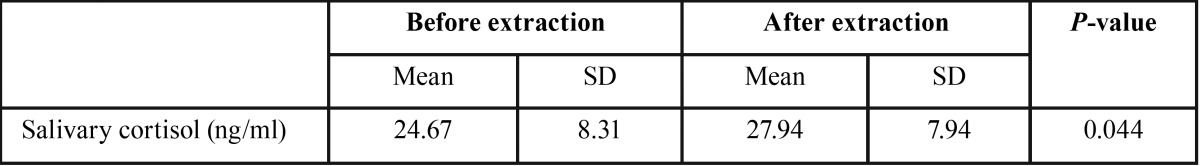
Comparison of salivary cortisol concentration before and after dental extraction in study group.

**Table 2:**
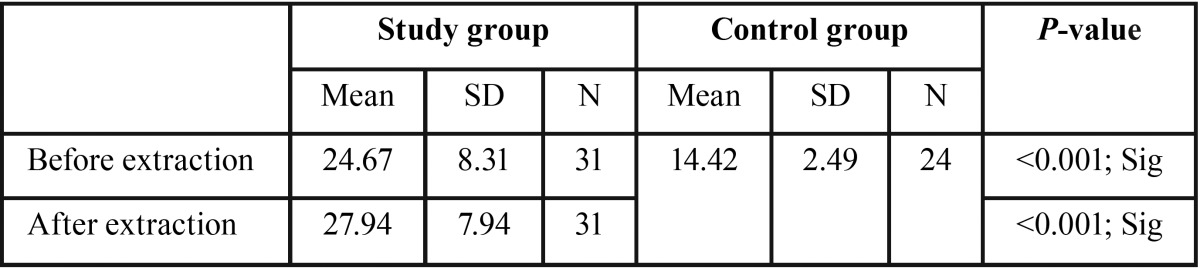
Comparison of salivary cortisol concentration before and after dental extraction with the control group.

**Table 3:**
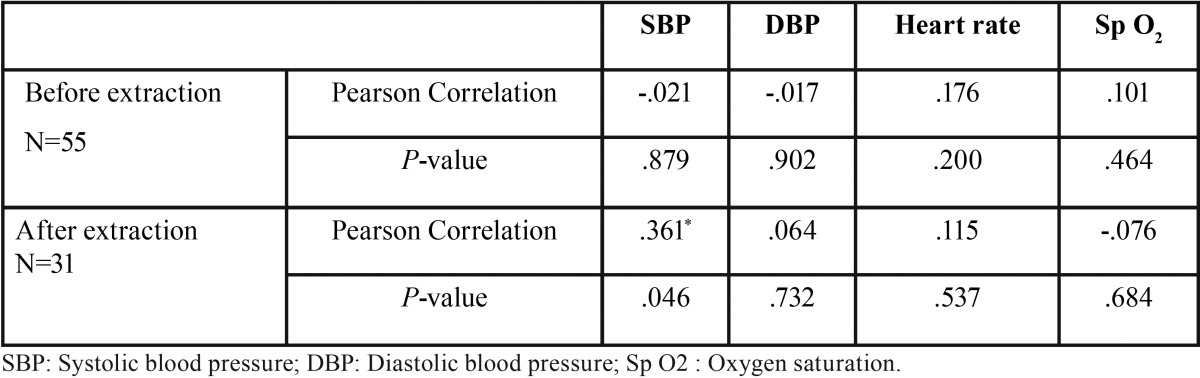
Correlation of salivary cortisol and hemodynamic parameters before and after dental extraction.

## Discussion

Anxiety, fear, and pain play crucial but often underemphasized in day to day dental practice. Physiologic stress in response to stimuli is a highly subjective emotion. Somatic changes induced by dental anxiety, may result from activation of the HPA axis. However, one of the most established methods to measure stress is by using cortisol (2). Cortisol is a potent glucocorticoid produced and secreted by the adrenal cortex, especially in response to stress via the activation of HPA axis. Previous studies have suggested that salivary cortisol correlates well with the free serum cortisol levels and therefore, provides the optimum method of collection of sample through a non-invasive technique (3).

The physiological response during an extraction or any other feared dental procedure is characterized by an initial increased heart rate and blood pressure, followed by a decreased heart rate and blood pressure. These changes can be attributed to the effect of sympathetic nervous system. In addition, stress and anxiety are a major cause of vasovagal syncope cases in a dental clinic and is commonly associated with dental extractions (9). For these reasons, it is imperative to observe changes in hemodynamics and oxygen saturation during a routine dental extraction to check for hypoxia.

The main aim of our study was to evaluate the change in salivary cortisol concentration during a dental extraction procedure and to assess if there is any correlation with hemodynamic parameters. No significant difference in the age of subjects was seen between the study and control group, and gender distribution was also not significant between the groups. Our study showed that in the study group, the level of salivary cortisol was higher after extraction when compared with before extraction levels. Also, the levels of the salivary cortisol before extraction in study group was significantly higher than the control group. This increased levels of salivary cortisol could be due to the stress in anticipation of the extraction and local anaesthetic injections. Studies previously conducted have shown that the dental procedures like dental prophylaxis, local anaesthetic injections, and extractions had elevated the levels of salivary cortisol (4-6). Our study showed a similar trend in the findings as well.

We could not establish any correlation or trend in the salivary cortisol levels and hemodynamic parameters, both before and after extractions. The hemodynamic parameters are influenced by systemic cortisol, which is present in serum and blood. Although there is a high correlation between salivary cortisol levels and unbound free cortisol levels in plasma and serum, there is a differential proportion of salivary versus total cortisol at lower and higher ranges. Due to this differential gradient, there may not be a linear trend between serum and salivary cortisol concentrations in response to a challenge (9). However, no studies were conducted on the relationship of hemodynamics and salivary cortisol. Gregg et al. concluded that salivary cortisol increased in response to stress but only weakly correlated with hemodynamic changes (10). They also suggested that the variations in the hemodynamics in anticipation, can last much longer than the actual stress itself. This could be one of the reasons for lack of correlation of salivary cortisol with hemodynamic parameters in our study. Agani *et al.* reported a significant correlation between extraction of teeth and hemodynamic changes including both the blood pressure and pulse rate, regardless of the presence or absence of a vasoconstrictor in the local anaesthesia. These findings have to be evaluated further in future studies due to inconsistent evidence (8).

To conclude, dental extractions and local anaesthetic procedures can induce stress in subjects. Dental care providers should try to minimise the subject’s anxiety and stress to the maximum extent. The dental surgeon should explore newer techniques to potentially decrease the stress and pain incurred to the patient. Lastly, the dental surgeon should follow surgical protocols diligently and perform dental extractions as efficiently as possible while minimizing any trauma, both physical and emotional. Salivary cortisol can be useful biomarkers to assess stress in subjects undergoing dental treatment. Development of chair side rapid evaluation salivary cortisol kits can help monitor stress among subjects undergoing dental treatment.
